# Mitochondrial DNA depletion syndrome and its cardiac complication

**DOI:** 10.3389/fcvm.2025.1582219

**Published:** 2025-06-10

**Authors:** Shengfang Bao, Jiajun Ye, Jiaqi Zhou, Chen Zheng, Yuejuan Xu, Sun Chen

**Affiliations:** ^1^ Department of Pediatric Cardiology, Xinhua Hospital, School of Medicine, Shanghai Jiao Tong University, Shanghai, China; ^2^ Department of Rheumatology and Immunology, Shanghai Children’s Medical Center, School of Medicine, Shanghai Jiao Tong University, Shanghai, China

**Keywords:** mitochondrial DNA depletion syndrome, cardiomyopathy, mtDNA replication, nucleotide metabolism, mitochondrial dynamics, mitochondrial damage, mitochondrial dysfunction

## Abstract

Mitochondrial depletion syndrome (MTDPS) is a heterogeneous group of genetic disorders characterized by a significant reduction in mitochondrial DNA (mtDNA) copy number, leading to the impaired mitochondrial function. The pathogenesis of MTDPS includes impaired mtDNA replication, damaged nucleotide metabolism and dysregulated mitochondrial dynamics. Due to its high energy demands, the heart is sensitive to the mitochondrial dysfunction. And the energy deficiency caused by the MTDPS contributes to the development of the mitochondrial cardiomyopathy. In this review, we summarize the cardiac phenotypes in the MTDPS, and the role of the mitochondrial injury in the myocardial damage. In specific, the association of the MTDPS-causing genes and their cardiac phenotypes are detailed. Moreover, the current treatment strategies for MTDPS are summarized. This review aims to integrate the current knowledge on the MTDPS and its cardiac phenotypes in order to provide insights for the further research and the clinic management.

## 1 Introduction

Mitochondrial DNA (mtDNA) depletion syndrome (MTDPS), a disorder caused by defects in the mtDNA maintenance, arises from disrupted nuclear genes that affect proteins crucial for the mtDNA synthesis and replication (1). This leads to the reduced mtDNA, the impaired respiratory chain and adenosine triphosphate (ATP) production, which causes the specific tissue and organ dysfunction.

Animal mtDNA has average about 16,000 base pairs in length, and human mtDNA has 16,569 base pairs and encodes 13 proteins (2). The mitochondrial genome consists of a noncoding region (NCR) that includes promoters, origins of the replication of the heavy strand and the light strand, three conserved repeats, and a termination sequence. It encodes 37 genes essential for the oxidative phosphorylation, tRNAs, and rRNAs. The cut-off level of the mtDNA amount for the MTDPS diagnosis has been set to 35%–40% of the amount in age-matched controls (3).

Mitochondrial diseases exhibit a wide phenotypic diversity because of the heteroplasmy and the threshold effect (1–3). The MTDPS also encompasses a wide range of autosomal dominant or recessive disorders and is typically classified into four forms: myopathic, encephalomyopathic, hepatocerebral, and neurogastrointestinal. The myopathic MTDPS is characterized by its impact on the muscle, mostly stemming from mutations in the TK2 gene. The encephalomyopathic MTDPS primarily involves the brain and muscles. The hepatopathic MTDPS affects the brain and liver, associated with mutations in genes such as DGUOK, POLG, or TWNK. Lastly, the neurogastrointestinal MTDPS is notable for its effects on the brain and gastrointestinal tract, also known as the mitochondrial neurogastrointestinal encephalomyopathy (MNGIE) and mainly caused by the mutations of the TYMP gene. To be noticed, studies have found that MTDPS could affect the heart and some mitochondrial cardiomyopathies could be fatal (4–13). The cardiac involvement most commonly occurs in myopathic and encephalomyopathic MTDPS, which we will elaborate separately in each section.

## 2 Cardiac involvements of the MTDPS

Mitochondria compose 30%–40% of cardiomyocyte volume and supply energy through oxidative phosphorylation (OXPHOS) (14, 15). As the heart is sensitive to mitochondrial dysfunction due to its high energy demands, the energy deficiency caused by the mtDNA depletion contributes to the development of the mitochondrial cardiomyopathy, characterized by the hypertrophic cardiomyopathy (HCM) or the dilated cardiomyopathy (DCM) (16, 17). Currently, pathogenic mutants of MTDPS-causing genes have also been identified in families with the idiopathic cardiomyopathy. However, the incidence of cardiac phenotypes varies among different mutants of MTDPS-causing genes (Table 1). For example, no cardiac manifestations have been reported in mutants of DGUOK (18, 19), based on searches in public databases including the OMIM, the InterVar, and the PubMed. The tissue-specific expression pattern of DGUOK may explain the phenomenon of the predominant hepatic and neurological involvement, but no cardiac phenotype. Other mutants, like POLG, were only reported in family cases. The following reasons may contribute to the scarce presentation of cardiac manifestations in these types of the MTDPS. First, the embryonic lethality observed in homozygous MTDPS mouse models could potentially explain the low incidence of cardiac phenotypes observed in human MTDPS patients, as affected embryos might not survive. This phenomenon was observed in homozygous knockout mouse embryos including *POLG*^*−/−*^ (20) and *TFAM*^*−/−*^ (21). This pattern of the embryonic death increases the difficulty of studying the pathogenic mechanisms of these specific types of MTDPS, especially in the embryonic period. The impact of MTDPS related pathogenic genes in embryos has also been reported in human. Recently, there have been reports of prenatal-onset cases of MTDPS13 in the population, accompanied by ventriculomegaly, cardiac anomalies, and etc. (22). Secondly, the cardiac involvement in MTDPS patients may also be underdiagnosed due to early mortality from neurologic or hepatic failure. Additionally, the propensity in some existing MTDPS animal models to develop cardiac phenotypes, which were less commonly reported in corresponding human cohorts may arise from interspecies differences in species as well (23).

**Table 1:** Mitochondrial DNA depletion syndrome and its related cardiac involvements.

Gene	Protein	Protein Function/Pathway	Type of MTDPS	Clinical Features	Type of Inheritance	Type of mtDNA Aberration	Cardiac involvements in human	Cardiac involvements in mice	Reference	
									Human	Animal
mtDNA replication										
*POLG*	DNA polymerase gamma	Polymerase	Mitochondrial DNA Depletion Syndrome 4A/Hepatopathic form	Alpers–Huttenlocher syndrome/ataxia/PEO	AR/AD	D/MD/PM	ACM	Cardiac hypertrophy, cardiomegaly, biventricular dilation, and bradycardia	(62)	(57–60)
*TWNK*	Twinkle	Helicase	Mitochondrial DNA Depletion Syndrome 7	Perrault syndrome/PEO/ataxia/encephalopathy/IOSCA	AD/AR	D/MD/PM	In 24% (8/33) of the patients, cardiac abnormalities were reported including sinus bradycardia, DCM and atrial arrhythmias. Other cases: CAVB/idiopathic left ventricular hypertrophy/LBBB	In tissue-specific (heart and skeletal muscle) Twinkle knockout mice, generated by crossing *Twinkle^+^^/^^loxP^* mice with Ckmm-cre mice, progressive heart enlargement consistent with mitochondrial cardiomyopathy was presented from 8 weeks. Homozygous knockout mouse embryos (*TWNK*^−/−^) die at ∼E8.5 due to severe mtDNA depletion.	(54–56)	(53)
*TOP1MT*	mitochondrial type IB topoisomerase	Topoisomerase	Mitochondrial DNA Depletion Syndrome 15	Mitochondrial complex I deficiency, nuclear type 33	AR	defective mtDNA regeneration following mtDNA depletion induced by ethidium bromide	HCM	TOP1MT knockout (KO) mice were susceptible to doxorubicin—induced cardiotoxicity.	(70)	(69)
*TOP3A*	DNA topoisomerase 3 alpha	Topoisomerase	/	PEO/Bloom syndrome-like disorder	AR	MD/D	rapidly progressed DCM/HCM	/	(67)	/
Nucleotide metabolism										
*TK2*	Thymidine kinase 2	dNTP anabolism	Mitochondrial DNA Depletion Syndrome 2/Myopathic form	Myopathy/PEO	AR	D/MD	One-third of early-onset patients (age ≤1 year) have extraskeletal muscle manifestations including cardiomyopathy, while less than 20% of the childhood-onset (age > 1 through 12 years) show prolonged QT and arrhythmia.	*Tk2*^−^*^/^*^−^ animals showed mtDNA depletion. Depletion of mtDNA was most prominent in brain, less in heart (20% of controls).	(77–79)	(75, 76)
Mitochondrial dynamics										
*OPA1*	Dynamin-like 120 kDa protein, mitochondrial	GTPase/mitochondrial fusion	Mitochondrial DNA Depletion Syndrome 14	Optic atrophy/Behr Syndrome	AD	MD	Family reports: HCM/Early-onset myocardial infarction/Symptomatic tachycardia	DCM and heart failure	(46, 47, 85)	(86)
*AGK*	Acylglycerol kinase	Lipid metabolism	Mitochondrial DNA Depletion Syndrome 10	Congenital cataract/HCM/skeletal myopathy and lactic acidosis/Sengers syndrome	AD	D	Ten cases with AGK mutants identified in 12 cases of Sengers syndrome, characterized by HCM. Terminal heart failure is universally inevitable. Other case reports: DCM/LVNC	/	(87, 89, 90)	/
Other pathways										
*FBXL4*	F-box/LRR-repeat protein 4	Protein homeostasis	Mitochondrial DNA Depletion Syndrome 13	Encephalo-myopathy	AR	D	Cardiac involvements observed in 54% of cases include cardiomyopathy, congenital heart malformations, arrhythmia, and pulmonary hypertension.	Transfection of FBXL4 rescued the cardiac geometry and the mitochondrial integrity with altered mitochondrial dynamics in the adult mice model of the heart failure with preserved ejection fraction.	(95–97)	(94)
*SLC25A4*	Adenine nucleotide translocator	ADP/ATP carrier	Mitochondrial DNA Depletion Syndrome 12	PEO/cardiomyopathy/myopathy	AD/AR	MD	The cardiomyopathy was observed in 77% of cases (17/22), though most types were not differentiated.	*SLC25A4*^−*/*−^ mice develop cardiomyopathy and myopathy.	(48, 109)	(105, 108)
CHCHD10	coiled-coil-helix-coiled-coil-helix domain-containing protein 10	local and global stress responses	/	IMMD,ALS, FTD, SMAJ	AD	MD	Family report: Cardiomyopathy	Mitochondrial cardiomyopathy	(101)	(98–104)

D, depletion; MD, multiple deletions; PM, point mutations; ACM, arrhythmogenic cardiomyopathy; CAVB, complete atrioventricular block; DM, dilated cardiomyopathy; HM, hypertrophic cardiomyopathy; AD, autosomal dominant; AR, autosomal recessive; dNTP, deoxyribonucleoside triphosphate; IOSCA, infantile onset spinocerebellar ataxia; MNGIE, mitochondrial neurogastrointestinal encephalomyopathy; PEO, progressive external ophthalmoplegia; PM, mtDNA somatic point mutations; IMMD, autosomal dominant mitochondrial myopathy; ALS, amyotrophic lateral sclerosis; FTD, frontotemporal dementia; SMAJ, late-onset spinal muscular atrophy.

## 3 Role of mitochondria in cardiac development and homeostasis

Mitochondrial function is essential for cardiomyocytes. The distribution and metabolic function of these organelles varies depending on the developmental stage of the myocardium (24). In neonatal cardiomyocytes, the heart preferentially obtains its energy from glycolysis and glucose oxidation. During this period, mitochondria manifest a characteristic reticular distribution in the cytosol that allows them to move freely. As cardiomyocytes reach the state of the terminal differentiation, fatty acid beta-oxidation becomes a primary energy source. In adult cardiomyocytes, energy derives mainly from the oxidation of fatty acids, and the motility of mitochondria is restricted (25). To be noticed, in the cardiac hypertrophy and the heart failure, the cardiac energy metabolism undergoes a shift towards a phenotype similar to the fetal state (26, 27). This transformation is characterized by a reduction in mitochondrial fatty acid beta-oxidation and an elevation in glycolysis.

Apart from the mitochondrial related metabolism, mitochondrial morphology has been found acting as a mechanism for bioenergetic adaptation during cardiac pathological remodeling. Mitofusins, namely Mfn-1 and Mfn-2, are regulators of mitochondrial network. Double-knockout (DKO) mice of Mfn-1 and Mfn-2 develop DCM with reduced mitochondria biogenesis genes and mtDNA (28).

Mitochondrial abnormalities could activate inflammatory pathways as well. Pathological conditions of the unregulated and elevated reactive oxygen species (ROS) production result in oxidative stress through the oxidative damage to DNA and the cell death (29, 30). Dysregulated ROS production and oxidative stress have been implicated in a host of cardiac diseases (31). ROS has been found as a signaling molecule to activate signaling pathways associated with inflammation, such as the NF-κB pathway, to further promote the generation of inflammatory mediators. The mtDNA has been found to be released through minority mitochondrial outer membrane permeabilization (miMOMP) (32–34). The oxidized mitochondrial DNA, which escapes from mitochondria, could ignite the NLRP3 and the cGAS-STING signaling (35). In heart failure, the cGAS-STING signaling is progressively activated, leading to the pathological cardiac remodeling and left ventricular dysfunction (36). These results suggest that the cytosolic release of mtDNA harms the cardiac function by activating inflammatory pathways. The cGAS-STING signaling has also been identified in the mutants of TFAM and TOP1MT related MTDPS, separately (37, 38). Moreover, *TFAM ^+^*^*/*−^ mice exposed to ionizing radiation exhibit enhanced nDNA repair responses in spleen (39). It can be inferred that subsequent release of mtDNA elicits a protective signaling response that enhances nDNA repair in cells and tissues, suggesting mtDNA is a genotoxic stress sentinel (39). In general, the effect of the release of mtDNA on the nDNA repair remains further study. Whether the activation of the cGAS-STING plays a part in the cardiac involvements of the MTDPS needs further elucidation.

## 4 Genes involved in the etiology of MTDPS with cardiac phenotypes

The MTDPS is one of the subgroups of the mtDNA maintenance disorder (MMD) and the other subgroup is the mtDNA multiple deletion syndrome (40). More deleterious and often recessive variants usually cause the mtDNA depletion, leading to early-onset, severe, multisystemic, and potentially fatal conditions. On the other hand, less severe and often dominant variants result in more slowly progressive, adult-onset myopathic diseases characterized by the accumulation of multiple mtDNA deletions (41). However, recent research showed that both depletion and multiple deletions could appear in the one patient at the same time, indicating they may stem from the same genetic defect (42, 43).

**Figure 1:**
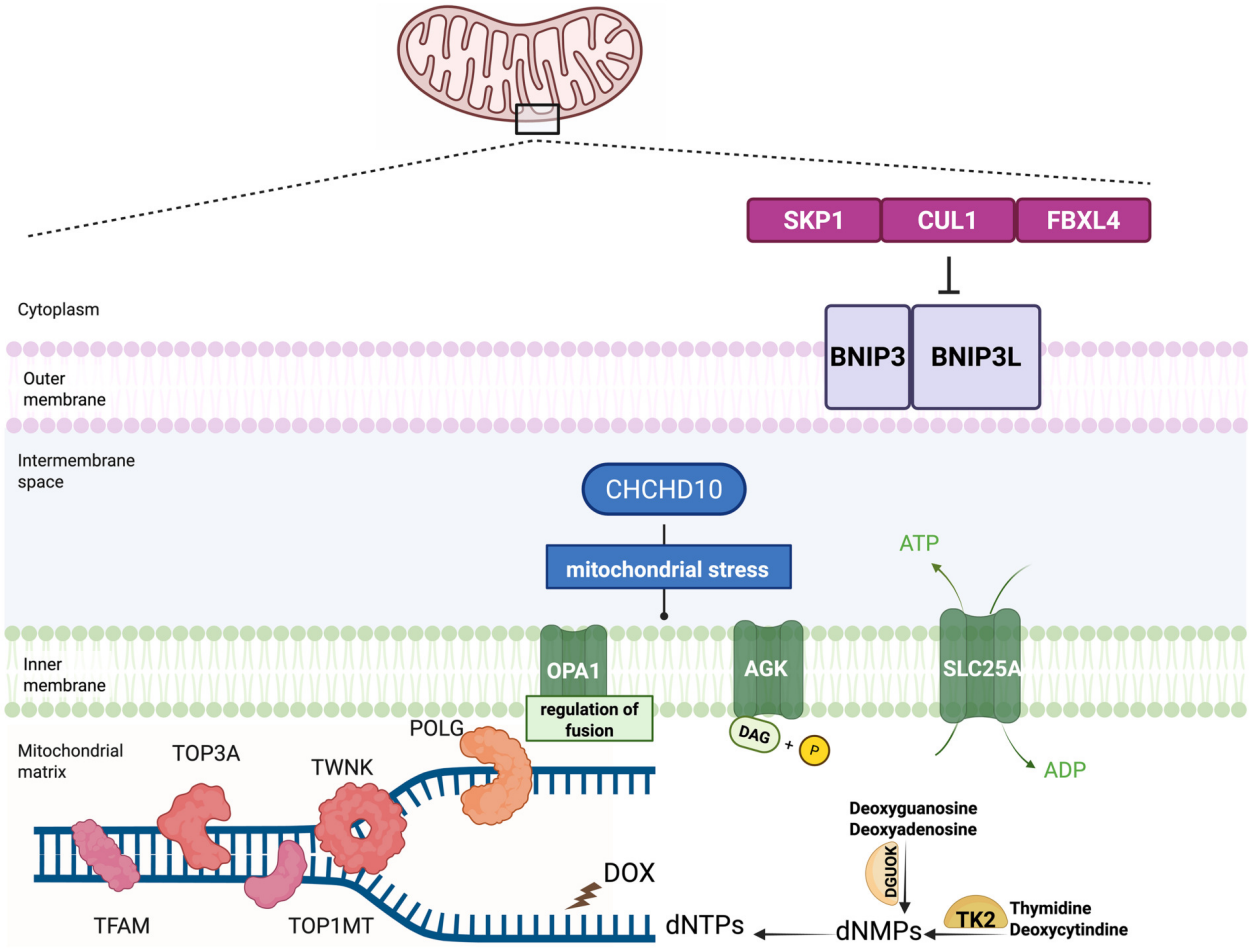
Model of pathogenesis involved in the mitochondrial depletion syndrome and the circumstance of doxorubicin mimicking the mitochondrial depletion syndrome. Dysfunctional mtDNA maintenance, nucleotide imbalance, and mitochondrial dynamics contribute to the mitochondrial depletion syndrome. The crucial proteins participating in the mitochondrial DNA maintenance include POLG, TWNK, TFAM, TOP3A and TOP1MT. Nucleotide metabolism plays a crucial role in the development of the MTDPS as well. Two key enzymes in the nucleotide salvage pathway, TK2 and DGUOK, are responsible for maintaining the mitochondrial dNTP pools. Mitochondrial dynamics are essential for maintaining the mitochondrial function. The proteins participating in the mitochondrial fusion, fission, and transport include OPA1 and AGK. FBXL4 serves as a component of E3 ubiquitin ligase complexes, called SKP1-CUL1-F-box (SCF) complexes. The SCF-FBXL4 E3 ubiquitin ligase complexes function as the suppressor of the mitophagy by mediating the degradation of BNIP3 and BNIP3l mitophagy receptors. FBXL4 functions to protect mitochondria from the depletion by inhibiting the mitophagy. Doxorubicin (DOX), widely used in oncology, could disrupt mitochondrial homeostasis by depleting mtDNA mimicking MTDPS.

The pathogenesis of MTDPS can be classified into four categories according to the biological process:(1) mtDNA replication; (2) deoxyribonucleotide metabolism; (3) mitochondrial dynamics; and (4) other functions (44–48) (Table 1) (Figure 1).

### 4.1 Pathogenesis involved in the mtDNA replication

The crucial proteins participating in the mitochondrial DNA maintenance comprise mitochondrial single-strand binding protein (MTSSB), which stabilizes unwound DNA, and the Twinkle protein (TWNK), which unwinds the double helix before replication at the origin of replication (OriH). Mitochondrial DNA-directed RNA polymerase (POLRMT) initiates RNA primer synthesis, while DNA polymerase gamma (POLG) extends DNA polymerization (1). These mtDNA replication and maintenance proteins play critical roles in the pathogenesis of MTDPS.

#### 4.1.1 TWNK

TWNK, located at the 10q24.31, encodes the mitochondrial helicase Twinkle in the mitochondrial nucleoids, and is crucial for the mtDNA replication and maintenance. TWNK unwinds the double helix of the DNA to initiate the replication (49, 50).

TWNK consists of a 5-primase domain, a linker region, and a helicase domain. Most pathogenic mutations are related to the linker region and the helicase domain. Dominant TWNK mutations cause the adult-onset progressive external ophthalmoplegia (PEO) and the proximal weakness (51). Recessive TWNK mutations lead to the MTDPS 7, manifesting as severe neurological disorders, the liver disease, the epilepsy, and the infant death (52).

Homozygous knockout mouse embryos (*TWNK*^*−/−*^) died at ∼E8.5 due to severe mtDNA depletion. In tissue-specific (heart and skeletal muscle) TWNK knockout mice, generated by crossing *TWNK*^+*/loxP*^ mice with Ckmm-cre mice, progressive heart enlargement consistent with mitochondrial cardiomyopathy was presented from 8 weeks. Severe mtDNA depletion and profound respiratory chain deficiency were observed in these mice (53).

In human, approximately 24% (8/33) of the patients harboring TWNK mutations, manifest cardiac abnormalities, including sinus bradycardia, cardiomyopathy and atrial arrhythmias (54, 55). Recently, the c.1485-1 G > A(p.T496_R531del) TWNK variant, resulting in a truncated mutant with part of the helicase domain, was identified in an autopsy case, aged 79-year-old, with complete atrioventricular block and idiopathic left ventricular hypertrophy (56).

#### 4.1.2 POLG

POLG, located at the 15q25, is a nuclear gene coding for the catalytic subunit of mitochondrial DNA polymerase, which exhibit a DNA polymerase activity, a 3′-5′ exonuclease activity that proofreads mis-incorporated nucleotides, and a 5′ deoxyribose phosphate lyase activity required for base excision repair.

In animal models, the POLG mutator mice carrying a proofreading-deficient form POLG-D257A point mutation results in reduced mitochondrial DNA integrity. These POLG-D257A mice exhibited a premature aging phenotype, though the degree of the cardiac dysfunction varied across studies (57–59). To figure out the influence on the heart, a cardiac targeted mouse model with POLG-Y955C was made and the cardiomegaly, biventricular dilation, and bradycardia were observed (60). The mechanism linking the premature aging and the cardiac involvement is not yet clear.

In human, POLG disease has been reported to be the most common single gene mitochondrial disease (10%) in the Australian adult population (61). Dominant POLG mutations manifest as slow progressive diseases in adults, typically PEO, ptosis, and myopathy (61). Recessive POLG mutations lead to MTDPS 4, manifested in the early life as multisystem and rapidly progressing neurodegenerative diseases, often accompanied by refractory epilepsy and end-stage liver failure (61). It was reported that a specific POLG mutation (c.2492A > G, p.Tyr831Cys) lead to the arrhythmogenic cardiomyopathy in a South African family (62). It seems POLG mutations play a role in the development of cardiomyopathy, although the precise pathogenesis requires further elucidation.

#### 4.1.3 TOP3A

The type IA topoisomerase 3*α* (TOP3A) removes the negative supercoiling and decatenate interlinked molecules (63). This decatenation activity of TOP3A is essential for separating replicated mtDNA molecules (63). Knockdown of TOP3A with siRNA could stall the replication fork, increase the mtDNA catenation and decrease the mtDNA copy number (64). To be noticed, TOP3A localizes in both the mitochondria and the nucleus. The function of TOP3A is complicated by the dual presence of mitochondrial and nuclear isoforms. The mitochondrial isoform of TOP3A has been found involved not only in the mtDNA segregation, but also the progression of the replication fork (64).

Recessive TOP3A variants can cause either mtDNA depletion and severe multisystemic neonatal disease, or adult-onset PEO, ptosis, and proximal myopathy, accompanied by peripheral neuropathy and cardiac disease (65). More severe variants result in a Bloom syndrome-like disorder (66).

Case-reports have demonstrated the involvement of TOP3A in both the DCM and the HCM, suggesting a complicated pathogenesis (67). Diverse manifestations of patients may be related with the phenomenon that TOP3A variants have different levels of impact on its functions. For example, the p.Leu37Val and p.Met575Val variants of TOP3A exhibited a significant loss of DNA-binding activity. While the p.Ser810* truncating variant showed a comparable level of DNA-binding activity (68). Another hypothesis of variable clinical presentations and different onset ages with similar genotypes is the influence of unidentified modifying variants.

#### 4.1.4 TOP1MT

Mitochondrial topoisomerase type IB (TOP1MT) is important for mtDNA regulation and is involved in mitochondrial replication, transcription, and translation. The susceptibility of TOP1MT knockout (KO) mice to doxorubicin-induced cardiotoxicity also underscores the role of TOP1MT in maintaining cardiac function (69). In human, two TOP1MT variants (R198C and V338l) at a highly conserved site in the core domain located within the DNA binding barrel were identified in a newborn with HCM, which implied the role of the mitochondrial topoisomerase in the cardiac phenotype (70). These two variants contributed to the mitochondrial dysfunction, including the impaired topoisomerase activity, altered mtDNA replication, and reduced mitochondrial translation.

#### 4.1.5 TFAM

Mitochondrial transcription factor A, abbreviated as TFAM, is the major protein constituent of the mammalian nucleoid. TFAM belongs to the high-mobility group domain proteins and induces a dramatic U-turn with an overall bend of 180° when bound to promoters or unspecific DNA (71, 72). In mouse models, heterozygous mice demonstrated a decrease in mtDNA copy number and a deficiency in the respiratory chain within the heart (21, 73). Furthermore, a potential pathogenic link between the TFAM dysfunction and the doxorubicin-induced cardiotoxicity was found (74). The embryonic lethality was reported in mouse model with *TFAM*^*−/−*^ (21). In human, homozygous or compound heterozygous mutations in the TFAM gene cause MTDPS 15, characterized by the onset of severe progressive liver disease soon after birth. Cardiac phenotypes associated with TFAM mutations have not yet been identified.

### 4.2 Pathogenesis involved in the nucleotide metabolism

Nucleotide metabolism plays a crucial role in the development of the MTDPS. Two key enzymes in the nucleotide salvage pathway, thymidine kinase 2 (TK2) and deoxyguanosine kinase (DGUOK), are responsible for maintaining the mitochondrial deoxynucleotide triphosphate (dNTP) pools.

The TK2 regulates the first and the rate limiting step in the phosphorylation of deoxypyrimidine nucleosides in the mitochondria. Mutations in TK2 manifests in a range of clinical symptoms, with myopathy being the most frequently observed. Specifically, skeletal muscle contains TK2 levels that are 5- to 14-fold lower than those in the liver, heart, and fibroblasts. This relative deficiency may render skeletal muscle more vulnerable to TK2 dysfunction (75).

The currently available TK2 animal models cannot definitively establish a link between TK2 and the cardiac phenotype because the homozygous TK2 mutant mice developed the progressive weakness and the tremor shortly after birth and died at the early age of 2–3 weeks before the possible appearance of the cardiac phenotype (76). In order to figure out the impact of TK2 on the cardiac phenotype, the cardiomyocyte or the heart-specific Cre mouse models are needed to further elucidate the mechanisms underlying cardiomyopathy caused by TK2 mutations.

It was reported that one-third of early-onset patients with the TK2 mutation(age ≤1 year) had extraskeletal muscle manifestations including cardiomyopathy, while less than 20% of the childhood-onset (age > 1 through 12 years) patients showed prolonged QT and arrhythmia (77). Three cases with cardiac manifestations were reported, including cardiac arrest caused by the ventricular fibrillation (78). The other cases with fatal mitochondrial HCM were observed, combined with the leucoencephalopathy and the hepatic steatosis at the age of 18 months (79).

### 4.3 Pathogenesis involved in the mitochondrial dynamics

Mitochondrial dynamics refers to the continuous processes of mitochondrial fusion, fission, and transport that are essential for maintaining the mitochondrial function and the cellular homeostasis. Disruptions in these processes can significantly contribute to diseases.

#### 4.3.1 OPA1

Optic atrophy 1 (OPA1) encodes a dynamin-like GTPase protein in the inner mitochondrial membrane (80). It involves in the regulation of the mitochondrial fusion, the cristae structure, the stability of the respiratory chain, and the mtDNA maintenance (81). The mitochondrial peptidases YME1l and OMA1 process OPA1 (69). In mouse models, cardiac-specific ablation of YME1l activated OMA1, accelerating OPA1 proteolysis, which induced mitochondrial fragmentation and altered cardiac metabolism, leading to DCM and heart failure (69). In return, the deletion of OMA1 prevented OPA1 cleavage, rescuing cardiac function and restoring mitochondrial morphology. Additionally, interventions of a high-fat diet or skeletal muscle-specific YME1l ablation were shown to restore cardiac metabolism and preserve heart function, independent of mitochondrial fragmentation suppression. Thus, OPA1 plays a critical role in sustaining cardiomyocyte survival. Intriguingly, cardiac metabolism and mitochondrial morphology are closely interconnected. Recent research has further revealed that mitochondria can be divided into two distinct functional subpopulations based on their reliance on OXPHOS-dependent ATP production (82). This discovery not only enriches but also complicates the understanding of mitochondrial morphology and metabolic regulation.

In human, mutations in the catalytic GTPase domain of OPA1 were linked to dominant optic atrophy (83). OPA1 also plays a pivotal role in the formation and function of cardiomyocytes (84). A homozygous missense mutation in the OPA1 gene (c.1601T > G, p.Leu534Arg) was identified in siblings from a consanguineous family. This mutation resulted in severe clinical manifestations, including lethal infantile-onset encephalopathy, optic atrophy, and HCM (85). In these siblings, a significant reduction in OPA1 protein levels was observed, attributed to the structural disturbances of the GTPase domain. Muscle biopsies revealed the mtDNA depletion (85), consistent with findings in OPA1-mutated mouse models (86). Other reports of cardiac manifestations in families with heterozygous OPA1 mutations include early-onset myocardial infarction (46) and symptomatic tachycardia (47). These observed symptomatic and even fatal cardiac manifestations extend the phenotype associated with pathogenic OPA1 mutations. Although cardiac manifestations are currently documented only in familial cases, cardiac monitoring is still warranted for patients with dominant optic atrophy.

#### 4.3.2 AGK

Acylglycerol kinase (AGK) plays a role in mitochondrial lipid metabolism and dynamics. AGK catalyzes the phosphorylation of diacylglycerol (DAG) using ATP and acylglycerol as substrates, producing ADP and acylsn-glycerol 3-phosphate as byproducts. Located in the inner mitochondrial membrane, AGK functions as a multisubstrate lipid kinase, and its activity triggers ROS signaling through a pathway involving protein kinase D1 (87).

The underlying pathogenic mechanisms of these cardiac phenotypes are intricate. Besides its role as a lipid kinase, AGK is an integral component of the mitochondrial translocase of the inner membrane 22 (TIM22) complex. This complex is essential for the translocation of transmembrane proteins into the mitochondrial matrix. Mutations in AGK that disrupt this function can compromise mitochondrial membrane integrity and impair energy production. Studies using AGK knockout cells have revealed downregulation of mitochondrial carrier proteins, OXPHOS components, and enzymes involved in mitochondrial one-carbon (1C) metabolism (88). As a result, cardiac energy metabolism is severely impaired, ultimately resulting in the cardiomyopathy and the heart failure. These observations highlight the critical role of AGK in the mitochondrial function and its related cardiac phenotypes.

In human, autosomal recessive mutations in AGK are associated with Sengers syndrome (10 cases with AGK mutants identified in 12 cases of Sengers syndrome) (87), which was characterized by congenital cataracts, HCM, skeletal myopathy, and lactic acidosis. Terminal heart failure secondary to HCM is universally inevitable and leads to death (89). There were also cases of DCM and a single instance of left ventricular non-compaction cardiomyopathy(LVNC) (90).

### 4.4 Pathogenesis involved in other pathways

#### 4.4.1 FBXL4: mitophagy related

F-box and leucine-rich repeat protein 4 (FBXL4) encodes a member of the F-box protein family characterized by the F-box motif, which serves as a component of E3 ubiquitin ligase complexes, called SKP1-CUL1-F-box (SCF) complexes. The SCF-FBXL4 E3 ubiquitin ligase complexes at the mitochondrial outer membrane function as the suppressor of the mitophagy by mediating the degradation of the BCL2/adenovirus E1B 19 kDa interacting protein 3(BNIP3) and NIP3-like protein X(Nix)/BNIP3l mitophagy receptors (91). FBXL4's role in controlling mitophagy levels were observed in patient cells and animal models (92). It has been found that FBXL4 mutations impair the SCF-FBXL4 complex formation, and thus cause the excessive mitophagy (93). Transfection of FBXL4 rescued the cardiac geometry and the mitochondrial integrity with altered mitochondrial dynamics in the adult mice model of the heart failure with preserved ejection fraction (94).

In the FBXL4-related encephalomyopathy, also known as the MTDPS13, increased mitophagy and mitochondrial DNA depletion were identified in patient's fibroblasts (95). Among the MTDPS13, the cardiac involvement was observed in 54% of cases (20/37), with specific manifestations including cardiomyopathy (27%, 10/37), congenital heart malformations (19%, 7/37), arrhythmia (15%, 6/41), and pulmonary hypertension (11%, 4/37) (96). The typical cardiomyopathy is HCM (96). LVNC was also reported (97).

#### 4.4.2 CHCHD10: metabolic and stress sensor

Coiled-coil-helix-coiled-coil-helix domain containing protein 10(CHCHD10) enriches at cristae junctions within the mitochondrial intermembrane space. CHCHD10 restrains the initiation of the mitochondrial integrated response stress, and suppresses the processing of OPA1 for mitochondrial fusion by interacting with OMA1 and suppressing its enzyme activity (98). A recent study on adipocytes and adipose tissue-specific CHCHD10 overexpressing mice has found that CHCHD10 is involved in cellular metabolic homeostasis as a metabolic sensor (99). Knock in mouse models bearing CHCHD10 variants were made and could present the symptoms of the CHCHD10-related disease including mitochondrial myopathy, cardiomyopathy, and amyotrophic lateral sclerosis (98–102). Specifically, in the CHCHD10-G58R knockin mice(C10G58R), complex I and complex IV activities were diminished, and multiple mtDNA deletions and decreased mtDNA copy numbers were observed in the heart (99–101). To be noticed, an extensive cardiac metabolic rewiring triggered by the proteotoxic mitochondrial stress response was identified in the C10G58R mice. The stress response began before the overt OXPHOS dysfunction (98). Specifically, the metabolic imbalance from the oxidative to the glycolytic metabolism and the activation of the 1C metabolism were triggered to provide increased 1C units for the methionine biosynthesis and transsulfuration. Meanwhile, increased NADPH oxidases elicit antioxidant responses. The metabolic stress response was believed to prolong the survival of C10G58R mice, though the fatal cardiomyopathy was inevitable as the disease progresses (103). Further studies were needed to elucidate the role of the mitochondrial stress response in the pathogenesis of MTDPS related cardiac phenotypes (104).

In human, due to the limited number of affected individuals reported so far and its recent discovery, the natural history of CHCHD10-related diseases is unclear (102). An autosomal dominant p.G58R mutation of CHCHD10 was reported in a family, presenting characteristics of mitochondrial myopathy and cardiomyopathy (101). The proband received a heart transplant due to the heart failure caused by his cardiomyopathy at the age of 20 (101).

#### 4.4.3 SLC25A4: mitochondrial carrier family

The Solute Carrier Family 25 Member 4 (SLC25A4) gene, which is responsible for encoding the heart- and muscle- specific isoform 1 of the adenine nucleotide transporter (ANT) located in the inner mitochondrial membrane (IMM), also known as ANT1, plays a critical role in transporting ADP into the mitochondrial matrix and ATP from the mitochondrial matrix into the intermembrane space. SLC25A4 also participated in the components of mitochondrial permeability transition pore governing *in vivo* cell death (105). Recently, SLC25A4 was identified as a direct target of S-nitrosoglutathione reductase, functioning to maintain mitochondrial homeostasis in hearts (106). The expression of SLC25A4 was found increased in hearts of the myocardial infarction and SLC25A4 was identified as a biomarker of the apoptosis-associated cardiomyocyte subcluster with single-cell data of from Gene Expression Omnibus database (107). These current findings demonstrated that SLC25A4 could be a potential novel therapeutic target for the heart failure. SLC25A4 knockout (*SLC25A4*^−*/*−^) mice develop cardiomyopathy and myopathy as well (108).

Patients carrying a SLC25A4 mutation manifest with encephalo-myo-cardiomyopathy. The cardiomyopathy was observed in 77% of cases (17/22), though the majority of the studies did not differentiate the types and not mention whether these patients combined with the arrhythmia (48). For example, a *de novo* dominant SLC25A4 mutant (c.239G > A, p.Arg80His) was reported in a female infant, who presented with HCM, hypotonia, elevated lactate levels. She eventually passed away at 14 days. Severe mitochondrial respiratory chain deficiencies, and the mitochondrial DNA depletion in skeletal muscle, caused by the impairment of SLC25A4 were found in this patient (109). In addition, the severity of SLC25A4 mutants was related with the mtDNA haplogroup (110). It was found that the mtDNA haplogroup U, which was defined by the mutations of A11467G, A12308G and G12372A, was linked to a more severe and rapidly progressive cardiomyopathy (110).

### 4.5 Cardiotoxicity of chemotherapeutic agents inducing mtDNA depletion

Emerging evidence highlights a critical intersection between mitochondrial genome integrity and chemotherapeutic toxicity. Anthracyclines (e.g., doxorubicin), widely used in oncology, exhibit dual roles. While inducing tumor cell death via nuclear DNA damage, they concurrently disrupt mitochondrial homeostasis by depleting mtDNA via the BAX/BAK-mediated mitochondrial membrane permeabilization and the cGAS-STING hyperactivation (111–113). This collateral mitochondrial toxicity poses heightened risks of myocardial dysfunction for patients whose mtDNA maintenance machinery were compromised. In detail, a negative correlation between the mitochondrial copy number and the heart failure biomarkers was observed in cancer patients' peripheral blood mononuclear cells after the anthracycline chemotherapy (113). Human induced pluripotent stem cells-derived cardiomyocytes with the pre-existing mtDNA depletion(mimicking MTDPS) showed the amplified doxorubicin-induced cardiotoxicity compared to controls, as mitochondrial insufficiency impairs the detoxification of ROS and augments the mtDNA leakage. It was reported that over expression of mitochondrial tagged DNase1 in mice could partially rescue doxorubicin-induced cardiac dysfunction (113). These insights offer new co-administration strategies for cancer patients with the compromised mitochondrial amount.

## 5 Treatment of mtDNA depletion syndrome

There are no efficacious treatments for MTDPS, so the management is mainly directed toward the symptomatic relief. For those MTDPS with cardiac involvements, heart failure in the MTDPS should be treated in the similar way as the heart failure triggered by other causes.

Administration of beta-blockers, angiotensin-converting enzyme inhibitors, neprilysin inhibitors, angiotensin-II receptor blockers, and diuretics may be given to restore the systolic and diastolic dysfunction. For patients with severe cardiac involvements, the heart transplantation is inevitable.

Recently, preclinical studies and clinical trials were involved including the nucleoside bypass therapy, stem cells and organ transplantation (114). Early recognition and immediate therapy to restore mitochondrial function might potentially improve clinical course.

### 5.1 Nucleoside bypass therapy

Clinical trials studies and *in vitro*/*in vivo* research studies showed that the enhancement of the salvage pathway by increasing the availability of deoxyribonucleosides needed for each specific genetic defect may prevent mtDNA depletion (115). Deoxycytidine and deoxythymidine (dC/dT) has been regarded as a treatment option for patients with POLG-related disorders with the evidence of the improved Newcastle Mitochondrial Disease Scale (NMDS) score and electroencephalography after the 6 months of treatment (116). Patients with TK2 deficiency, who treated with a similar compound, reported the improvement on the life quality as well (117). In a phase II Trial (NCT04802707), a mix of dC/dT will be used as the early treatment of MTDPS, including the mutations of following genes: POLG, TWNK, RRM2B, MPV17, SUCLA2, SUCLG1, and FBXL4. These trials demonstrate that dC/dT may have broader therapeutic potential for a range of different mitochondrial disorders. Long time follow-up studies are expected in the future.

### 5.2 Others

Unlike approaches for most mitochondrial diseases, potentially disease modifying therapies are available for MNGIE. Therapeutic options include the direct removal of metabolites by peritoneal dialysis, the substitution of the missing enzyme and the enzyme replacement by the allogeneic hematopoietic stem-cell transplantation (118). Nevertheless, no cases of heart involvements have been reported in the patients of the MNGIE so far, so whether the options for the MNGIE could be used in the other MTDPS patients with heart involvements still depends. Although some approaches could have a more general benefit, others may only treat specific mitochondrial disease.

## 6 Conclusion

MTDPS represents a significant challenge due to its impact on multiple organ systems, with cardiomyopathy being a critical and often life-threatening component. Understanding the genetic basis and pathophysiological mechanisms underlying MTDPS can help developing better diagnostic and therapeutic strategies in order to ultimately improve outcomes for affected individuals. Ongoing research into mitochondrial biology and genetics holds promise for future advancements in the management of these complex disorders.
